# Synthesis, Characterization, and Nanomedical Applications of Conjugates between Resorcinarene-Dendrimers and Ibuprofen

**DOI:** 10.3390/nano7070163

**Published:** 2017-06-30

**Authors:** Luis D. Pedro-Hernández, Elena Martínez-Klimova, Sandra Cortez-Maya, Sonia Mendoza-Cardozo, Teresa Ramírez-Ápan, Marcos Martínez-García

**Affiliations:** 1 Instituto de Química, Universidad Nacional Autónoma de México, Ciudad Universitaria, Circuito Exterior, Coyoacán C.P. 04510, México D.F., Mexico; daniel.pedro.herandez@gmail.com (L.D.P.-H.); sandracrtz@gmail.com (S.C.-M.); smc0810@gmail.com (S.M.-C.); mtrapan@yahoo.com.mx (T.R.-Á.); 2 Instituto de Fisiología Celular, Universidad Nacional Autónoma de México, Ciudad Universitaria, Circuito Exterior, Coyoacán C.P. 04510, México D.F., Mexico; eklimova@email.ifc.unam.mx

**Keywords:** ibuprofen, resorcinarene-dendrimers, anti-tumor activity, cytotoxicity

## Abstract

Ibuprofen has been reported to possess anticancer activity. In the present work, four ibuprofen conjugates of resorcinarene-Polyamidoamine PAMAM-dendrimers were synthesized with eight or 16 ibuprofen moieties. The ibuprofen was released from the dendrimers in a dependent manner. The drug-conjugated nanoresorcinarene-dendrimers showed higher cellular uptake than free ibuprofen. In vitro cytotoxicity studies were performed with free ibuprofen and with the synthesized conjugates in U251, PC-3, K-562, HCT-15, MCF-7, SKLU-1, and MDA U251 (human glioblastoma), PC-3 (human prostatic adenocarcinoma), K-562 (human chronic myelogenous leukemia cells), HCT-15 (human colorectal adenocarcinoma), MCF-7 (human mammary adenocarcinoma), SKLU-1 (human lung adenocarcinoma), and MDA-MB-231 (human mammary adenocarcinoma) cancer cell lines by different cytotoxicity assays. Ibuprofen conjugates of the first and second generations showed significant cytotoxic effects towards the human glioblastoma (U251) and human mammary adenocarcinoma (MCF-7, MDA) cell lines. Moreover, the ibuprofen conjugates improved cytotoxicity compared to free ibuprofen. Increased therapeutic efficacy was observed with specific ibuprofen conjugates of the second generation using low doses.

## 1. Introduction

2-(4-isobutylphenyl) propionic acid, well known as ibuprofen, is a non-steroidal anti-inflammatory drug (NSAID) used to treat inflammatory rheumatoid diseases and relieve acute pain. The use of this therapeutic agent is restricted due to its side effects such as stomach ulceration, bleeding, and perforation [1,2]. The terminal acidic group accentuates its effectiveness but also produces gastric ulceration [3,4]. Several studies have shown that ibuprofen has anticancer activity, specifically against gastric cancer cells [5], HT-29 and HCT-116 colon carcinoma cells [6,7], and prostate cancer cells [8,9]. 

Ibuprofen has been used as an anticancer drug by itself or in conjunction with drug carriers such as hydrogels [10], liposomes [11,12], dendrimers [13], and micelles [14]. Of these drug carriers, dendrimers are a promising delivery system for several drugs due to their low solubility, size, high biocompatibility, good efficiency in drug delivery, and therapeutic activity [15]. Dendrimers also exhibit considerably less cytotoxicity and permeation than the drug alone [16]. Studies on the mechanism of interaction between polyamidoamine PAMAM-dendrimers and cells show that the main internalization mechanism is the clathrin-mediated endocytosis pathway, where vesicles fuse with early endosomes following an acidification route that finishes in lysosomes [17]. We previously reported that resorcinarene-PAMAM dendrimers of the first- and second-generation conjugates of 5-aryl-1,4-benzodiazepines showed good anticancer activity [18]. In the present study, we report our new in vitro anticancer activity results using resorcinarene-dendrimers as carriers of ibuprofen. 

## 2. Materials and Methods

### 2.1. Materials

2-(4-Isobutylphenyl) propionic acid (ibuprofen), (98%, Sigma-Aldrich, St. Louis, MO, USA), Resorcinol (99%, Sigma-Aldrich, St. Louis, MO, USA), hydrocinnamaldehyde (90%, Sigma-Aldrich, St. Louis, MO, USA), dodecyl aldehyde (92%, Sigma-Aldrich), hydrochloric acid (36.5–38% Aldrich), ethylendiamine (Sigma-Aldrich, St. Louis, MO, USA), methyl bromoacetate (Sigma-Aldrich, St. Louis, MO, USA), and methyl acrylate (Sigma-Aldrich, St. Louis, MO, USA) were used without additional purification. Triton^™^ X-100 solution (Sigma-Aldrich, St. Louis, MO, USA), phosphate-buffered saline pH 7.4 (Sigma-Aldrich, St. Louis, MO, USA), and ethanol (Sigma-Aldrich) were used without additional drying. Tetrahydrofuran THF was dried with Na. *N*,*N*-Dimethylformamide (DMF, Sigma-Aldrich, St. Louis, MO, USA), and dimethylsulfoxide (DMSO) (99.8%, Sigma-Aldrich, St. Louis, MO, USA). 

### 2.2. Synthesis of Resorcinarene with Poliaminoamide Dendritic Arm Dendrimers

Resorcinarene macrocycles and PAMAM-dendritic arms were synthesized and characterized as reported by Cortez-Maya et al. [18].

### 2.3. Synthesis of Ibuprofen Conjugates of First and Second Generations

Resorcinarene-dendrimers (0.0525 mmol) were dissolved in methanol (40 mL) and heated at 80 °C. After 20 min, ibuprofen (0.63 mmol) was added in methanol. The mixture was stirred and heated at 120 °C for 24 h. The solvent was evaporated and the resulting solid was dissolved in methanol and precipitated by ethyl acetate EtOAc.

### 2.4. Characterization of the Conjugates

^1^H and ^13^C nuclear magnetic resonance NM spectra were recorded on a Varian Unity-300 MHz with tetramethylsilane (TMS) as an internal reference. Infrared (IR) spectra were measured on a Bruker FTIR tensor 27 Nicolet FT-SSX spectrophotometer (Bruker, Billerica, MA, USA). Elemental analysis was determined by Galbraith Laboratories, INC Knoxville. FAB+ mass spectra were taken on a JEOL JMS AX505 HA instrument (JEOL manufacturers, Tokyo, Japan). Electrospray mass spectra were taken on a Bruker Daltonic, Esquire 6000 (Bruker, Billerica, MA, USA). MALDI-TOF mass spectra were taken on a Bruker Omni FLEX (Bruker manufacturers, Billerica, MA, USA) using 9-nitroanthracene (9NA) as a matrix. The ultraviolet-visible UV-vis absorption spectra were obtained at room temperature with a Shimadzu 2401 PC spectrophotometer (Shimadzu manufacturers, Kyoto, Japan). 

### 2.5. Cell Lines

U251 (human glioblastoma), PC-3 (human prostatic adenocarcinoma), K-562 (human chronic myelogenous leukemia cells), HCT-15 (human colorectal adenocarcinoma), MCF-7 (human mammary adenocarcinoma), SKLU-1 (human lung adenocarcinoma), and MDA-MB-231 (human mammary adenocarcinoma) cell lines were supplied by the National Cancer Institute (Bethesda, MD, USA). Human gingival fibroblasts (HGF) were supplied by the National Cancer Institute (Bethesda, MD, USA). Each treatment was performed in heptaplicate wells. The IC_50_ values were estimated by fitting the inhibition data to a dose-dependent curve using a logistic derivative equation. 

### 2.6. Hydrolytic Release Studies

The hydrolysis of conjugates was studied at pH 7.4 (0.1 M phosphate-buffered saline (PBS)). All experiments were performed in triplicate along with controls. Four milligrams of each conjugate were added into 4 mL of preheated buffer solutions. All of the release solutions containing 1 mg/mL of the conjugate were stirred continuously and maintained at 37 °C. At different time intervals, samples were withdrawn and the reaction was stopped with a methanol solution of sodium salicylate (1.25 mM) and analyzed by UV and IR spectroscopies. 

### 2.7. In Vitro Cellular Uptake of the Conjugates of Ibuprofen

A Synergy hybrid technology HT Microplate Reader (Bio-Tek Instruments, Winooski, VT, USA) quantitatively determined the cellular uptake of ibuprofen-conjugated dendrimers. PC-3 cancer cells were seeded in a 96-well black plate at 10,000 cells/well and cultured. After 15 days, the cancer cells came to 65–80% confluence. The culture medium was replaced by Transport Buffer (pH 7.4). The PC-3 cancer cells were incubated with either ibuprofen conjugates or free ibuprofen at a concentration of 10 μM/mL for 6 h at 35 °C. After incubation, the suspension was removed and cells were washed four times with 50 μL cold phosphate-buffered saline solution (PBS (pH 7.4)). After that, 100 μL of 2% Triton X-100 in PBS were added to the wells to lyse the cells. The fluorescence intensity of each sample well was measured by a microplate reader calibrated with standard solutions of ibuprofen (0.1–50 μM/mL) in similar conditions. The excitation and emission wavelengths were 220 and 410 nm, respectively, for ibuprofen. Experiments were performed in triplicate. 

### 2.8. In Vitro Cytotoxicity Studies

Cytotoxicity assays were determined using the protein-binding dye sulforhodamine B (SRB) in microculture to measure cell growth, as described in the protocols established by the National Cancer Institute NCI (Monks et al., 1991) [19]. Conjugates of ibuprofen and resorcinarene-dendrimers were prepared as stock solutions at the concentration of 5–10 μM in 2% DMSO and added into culture medium immediately before use. Control cells were treated with 2% DMSO. Mean values of IC_50_ for each tested drug were calculated from at least three different experiments. For the assay with Human gingival fibroblast (HGF) cells, the tested compounds were dissolved in fresh culture medium with 2% DMSO to afford different concentrations (1, 10, 100, 750, and 1000 μmol/L).

## 3. Results

### 3.1. Synthesis of Ibuprofen–Resorcinarene-Dendrimer Conjugates

**Chart 1:**
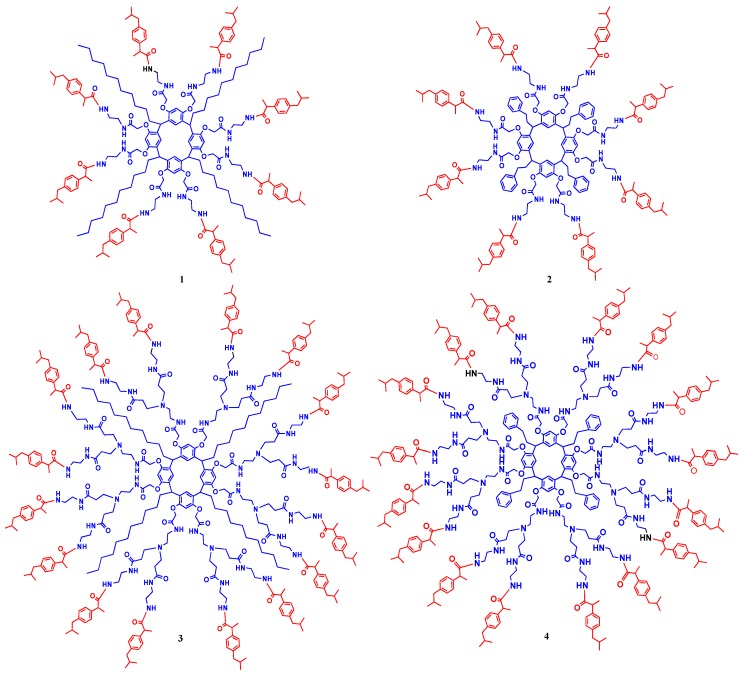
Resorcinarene-dendrimers of first- (**1**, **2**) and second-generation (**3**, **4**) conjugates of ibuprofen.

Recently, we showed that resorcinarene-dendrimers can be obtained with a crown conformation of first and second generations, with terminal amine groups that can be functionalized with the acid group of the ibuprofen to obtain the conjugates of first and second generations with eight and 16 ibuprofen moieties (Chart 1).

**Figure 1:**
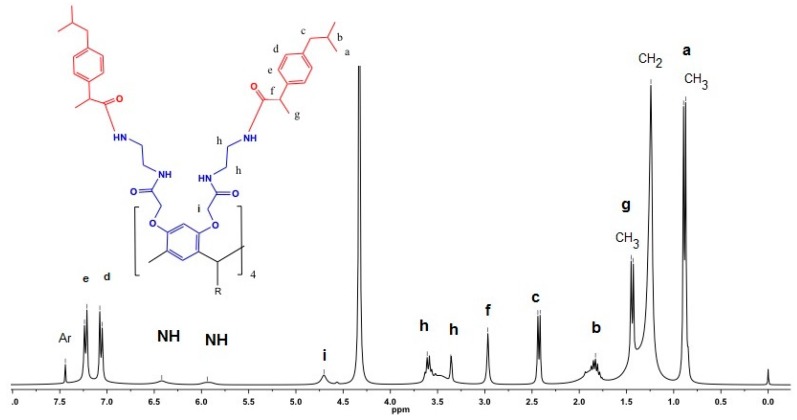
^1^H NMR spectrum of compound **1**.

Conjugate **1** was characterized by ^1^H NMR spectroscopy (Figure 1), and in the spectrum the most important signals are two doublets at δ_H_ 7.23 and 7.06 due to the *para* substituted phenyl at the ibuprofen with a coupling constant *J* = 7.8 Hz, *J* = 7.5 Hz, respectively, and two signals at δ_H_ 1.44 and 2.42 due to the isopropyl group at the ibuprofen (Full characterization of all the compounds see Supplementary Material). 

### 3.2. Hydrolytic Release of Ibuprofen and Ibuprofen Conjugates 

**Figure 2:**
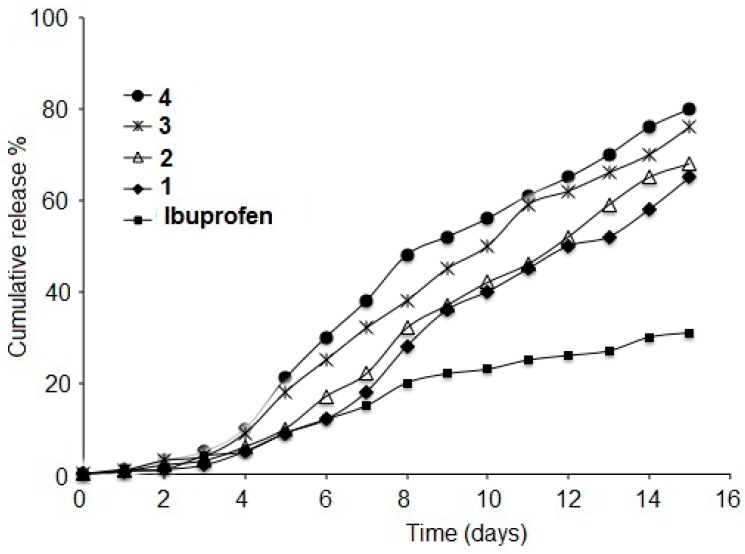
In vitro release profiles of ibuprofen from ibuprofen conjugates **1**–**4** at (pH 7.4) at 37 °C (mean ± SD, *n* = 3).

The in vitro hydrolysis behavior of ibuprofen and drug-dendrimer conjugates was studied in physiological conditions (phosphate-buffered saline (PBS), at 37 °C and pH 7.4). The dendrimers were dispersed in the buffer solution and hydrolysis was performed. The amount of free ibuprofen in the buffer solution was determined by UV (at 220 nm) and IR (at 1708 cm^−1^) spectroscopy. Detection of the hydrolyzing solution by UV spectrophotometry showed that only the ester bond between the drug moiety and the dendritic branches is hydrolyzed during the reaction time, and it was observed that the four ibuprofen-conjugated dendrimers **1**–**4** showed a relatively slow release in the first days followed by a sustained release in the second week. Ibuprofen was released from the corresponding first-generation **1**, **2** dendrimers at 68 and 65%, respectively, and the second-generation **3**, **4** dendrimers at 76 and 80% (Figure 2). The hydrolysis of drug-conjugate dendrimers depends on the length of the dendritic branches or the generation of the drug-dendrimer and the chemical structure of the dendrimer, which affect the release rate. 

### 3.3. The In Vitro Cellular Uptake of the Conjugates of Ibuprofen 

**Figure 3:**
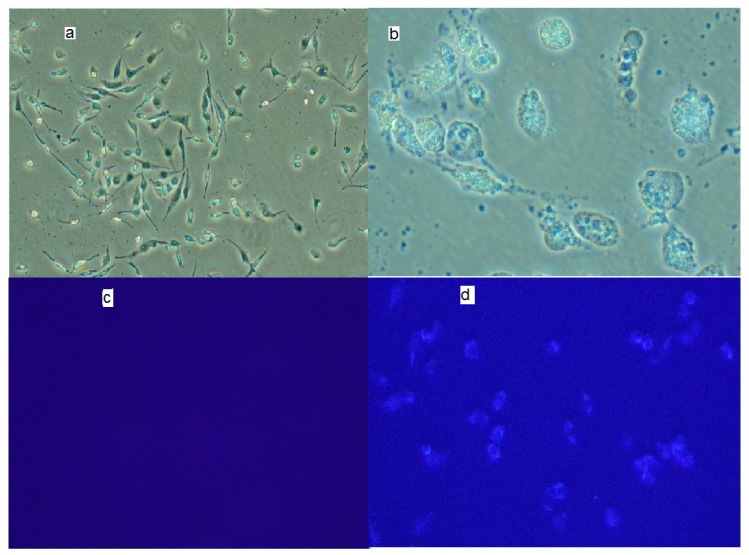
PC-3 morphology. (**a**) Control PC-3 cells (10×); (**b**) PC-3 cells with resorcinarene-PAMAM-ibuprofen dendrimer **4**; (**c**) No fluorescence of the PC-3 cells without conjugate is observed; and (**d**) fluorescence of the PC-3 cells incubated with resorcinarene-PAMAM-ibuprofen conjugate **4** (40× amplification).

The in vitro cellular uptake of the conjugates of ibuprofen in PC-3 cells was determined. PC-3 cells were incubated with ibuprofen and the dendrimer conjugates at a concentration of 10 μM for 6 h, before examination under a fluorescent microscope (10× and 40×). Dendrimer conjugates at concentrations of 10 μM were cytotoxic to PC-3 cells. Figure 3 shows the PC-3 control cells (Figure 3a). The control PC-3 cells without resorcinarene-PAMAM-ibuprofen did not show blue fluorescence (Figure 3c) after excitation with UV light. The PC-3 cells treated with resorcinarene-PAMAM-ibuprofen dendrimer **4** showed changes in morphology, and an increase in cell volume was also observed, resulting in more round and detached cells. The difference in the number of cells per plate was due to the inhibitory effect of conjugates on the proliferation rate (Figure 3b). The PC-3 cells with the dendrimer conjugate **4** showed blue fluorescence, documenting the uptake of the dendrimer conjugate (Figure 3d). It could be concluded from Figure 3d that the cellular internalization of resorcinarene-PAMAM-ibuprofen dendrimers could occur via clathrin-mediated endocytosis.

### 3.4. Cytotoxic Activity of Ibuprofen

**Figure 4:**
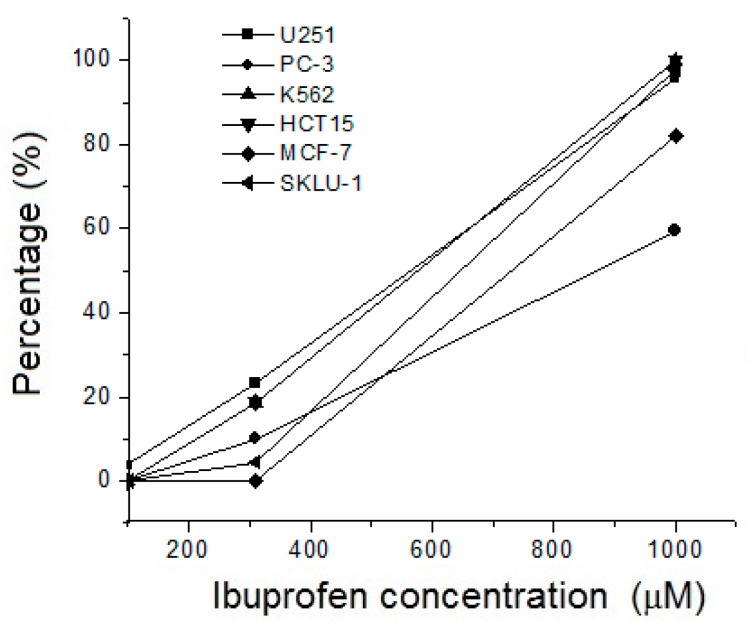
Cytotoxicity of ibuprofen against U251, PC-3, K-562, HCT-15, MCF-7, and SKLU-1 tumor cell lines. Cells were cultured in the presence of the indicated concentrations of ibuprofen for 72 h.

Recent reports showed that free ibuprofen is not toxic in low doses and has antitumoral effects [5,6,7,8]. To examine its activity, we first tested the effect of free ibuprofen in U251, PC-3, K-562, HCT-15, MCF-7, SKLU-1, and MDA-MB-231 cancer cells, at three different concentrations: 100, 310, and 1000 μM (Figure 4). It was observed that at all concentrations tested (100–1000 μM), free ibuprofen was cytotoxic for U251, K-562, HCT-15, and SKLU-1 cancer cells. Low cytotoxicity was observed for the PC-3 and MCF-7 cell lines at the highest concentration tested (1000 μM). This indicates that large concentrations of ibuprofen must be added to the PC-3 and MCF-7 cell lines to observe any cytotoxic activity. 

### 3.5. Cytotoxicity of Ibuprofen Conjugates

The cytotoxic activity of the synthesized resorcinarene-dendrimer-conjugates of ibuprofen **1**–**4** was evaluated. All compounds were chosen for evaluation of their biological activity against cancer. We screened in vitro against seven human cancer cell lines: U251 (human glioblastoma), PC-3 (human prostatic adenocarcinoma), K-562 (human chronic myelogenous leukemia cells), HCT-15 (human colorectal adenocarcinoma), MCF-7 (human mammary adenocarcinoma), SKLU-1 (human lung adenocarcinoma), and MDA-MB-231 (human mammary adenocarcinoma). As a control, we also tested against the human gingival fibroblast (HGF) cell line. The initially obtained cytotoxic screening data (Table 1) showed that at 10 μM, the dendrimer of first generation **1** showed high inhibition (%) of five cell lines, and the conjugate **2** showed high activity against three cell lines: PC-3, MCF-7-7, and MDA-MB-231. For the dendrimer of second generation **3**, the activity was 100% in five cell lines. The dendrimer **4** showed high specificity against the K-562 cell line. The antiproliferative results obtained with the conjugates of ibuprofen **1**–**4** were compared to ibuprofen on its own (free) and to cisplatin, a reference anticancer drug. 

**Figure 5:**
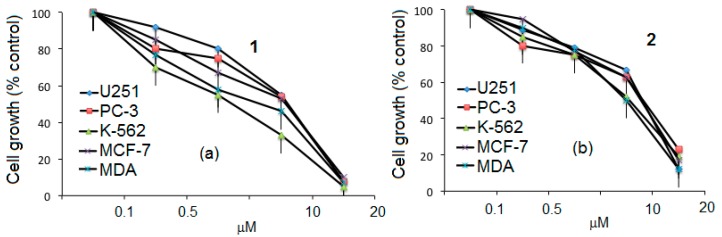

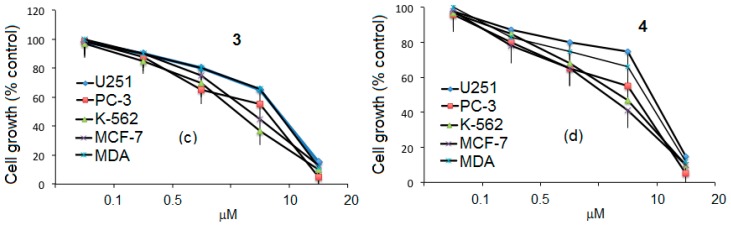
Dose–response curves obtained from U251, PC-3, K-562, MFC-7, and MDA cells exposed to compounds **1** (**a**); **2** (**b**); **3** (**c**); and **4** (**d**). Points represent mean values ± SD of seven independent experiments.

The antiproliferative experiments were carried out in order to gain some insight into the anticancer properties that are displayed in vitro. The cell lines were exposed for five days to different concentrations (from 0.1 to 20 μM) of the **1**–**4** ibuprofen conjugates. A marked dose-dependent inhibition of cell growth was consistently observed in all five cell lines. The detailed profile of the inhibition of these cell lines by our compounds is shown in Figure 5a–d. The five cell lines that were the most inhibited by compounds **1**–**4** were: U251 (human glioblastoma), PC-3 (human prostatic adenocarcinoma), K-562 (human chronic myelogenous leukemia cells), MFC-7 (human mammary adenocarcinoma), and MDA-MB-231 (human mammary adenocarcinoma). The dose-response behavior of the conjugates **1** and **3** with undecyl chains in the lower part of the resorcinarene showed that the activity depended on the amount of ibuprofen molecules in the dendrimer and the length of the dendritic arm. This also was observed with dendrimers **2** and **4**, which have ethylphenyl groups in the lower part of the resorcinarene. The second generation compound **4** was more active against all cell lines, even at low doses, than the first generation dendrimer **2**. 

**Figure 6:**
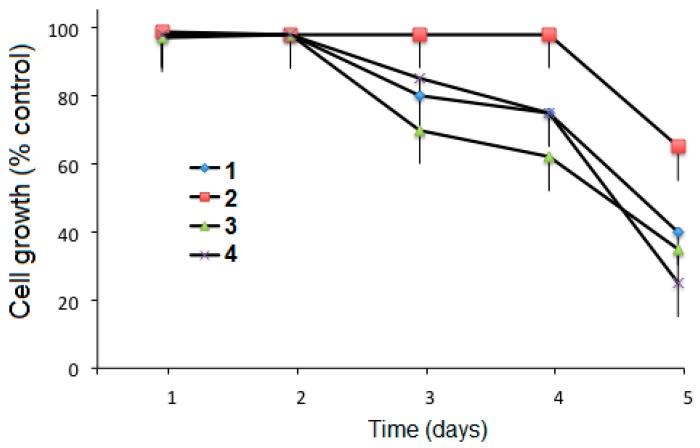
Cytotoxic activity of compounds **1**, **2**, **3**, and **4** for 5 days at 10 μM, against human gingival fibroblast (HGF) cells. Points represent mean values ± SD of seven independent experiments.

The cytotoxic activity of the conjugates **1**–**4** under the same conditions was tested against the human gingival fibroblast (HGF) cell line for five days (Figure 6). Compound **4** showed the lowest cytotoxic activity after five days. 

The inhibitory concentration (IC_50_) values determined for all cell lines (U251, PC-3, K562, MCF-7, SKLU-1, HCT-15, and MDA-MB-231) ranged from 0.1 to 5 μM with the conjugates of ibuprofen **1**–**4.** The results are shown in Table 2. In general, the in vitro experiments revealed the good anticancer activity of the tested compounds. The conjugate of first generation **1** had high activity against human glioblastoma (U251), human mammary adenocarcinoma (MCF-7), human lung adenocarcinoma (SKLU-1), and human mammary adenocarcinoma (MDA-MB-231). The conjugate **2** showed high selectivity against the human chronic myelogenous leukemia cells (K-562) and human mammary adenocarcinoma (MDA-MB-231) cell lines. The conjugate of second generation **3** was very active against all the cell lines studied. The conjugate **4** showed good activity against the five cell lines U251, K-562, MFC-7, SKLU-1, and MDA-MB-231. 

In particular, U251 human glioblastoma, K-562 human chronic myelogenous leukemia cells, MCF-7 human mammary adenocarcinoma, and MDA-MB-231 human mammary adenocarcinoma cancer cell lines appeared to be more sensitive to growth inhibition by the conjugates of ibuprofen than the PC-3, HCT-15, and SKLU-1 cancer cell lines. The most interesting result was obtained with compound **4**, which was found to be the best inhibitor of this series against U251 and K-562, MFC, and MDA cell lines with an IC_50_ of 3.3 ± 0.02, 3.0 ± 0.5, 3.0 ± 0.3, and 3.1 ± 0.5, respectively. Moreover, these compounds were tested against HGF normal fibroblasts in order to determine their selectivity for cancer cells compared to normal cells. Compound **4** showed higher selectivity for the cancer cells than for HGF fibroblasts. 

Four resorcinarene-dendrimer-conjugates of ibuprofen **1**–**4** were synthesized in this work: compound **1** had the corresponding dodecyl group in the lower part of the macrocycle and eight ibuprofen moieties in the structure. Compound **3** also had the corresponding dodecyl group in the lower part of the macrocycle and 16 ibuprofen moieties in the structure. With 16 moieties, compound **3** was more active than compound **1**. Compounds **2** and **4** had ethylphenyl groups in the resorcinarene macrocycle and had eight or 16 ibuprofen moieties, respectively. Compound **4** showed better results inhibiting cancer cell proliferation than compound **2**. The results of these experiments clearly indicate that **3** and **4**, with 16 ibuprofen moieties, inhibit the proliferation of all cancer cell lines tested. The ranked order of potency was **4** > **3** > **1** > **2** at the concentrations assayed.

## 4. Conclusions

In the present work, it was observed that the yields of synthesis to obtain the conjugates of ibuprofen with resorcinarene-dendrimers with aliphatic and aromatic substituents in the lower part of the macrocycle are generally good. The hydrolysis of drug-conjugate dendrimers depends on the length of the dendritic branches and the chemical structure of the dendrimer. These affect the release rate of ibuprofen. The cellular internalization of resorcinarene-PAMAM-ibuprofen dendrimers could occur via clathrin-mediated endocytosis. Biological activity tests showed that the synthesized compounds have high potential activity against cancer. The inhibition of cancer cell proliferation was enhanced in the presence of eight and 16 ibuprofen-moiety-substituents in the dendritic branches compared to ibuprofen alone. The conjugates were slightly cytotoxic to human gingival fibroblast cells and showed improved efficacy over the free drug. 

## Figures and Tables

**Table 1:** Inhibition on the growth (%) of human tumor cell lines by conjugates **1**–**4** at 10 μM in DMSO.

Cell Lines								
Sample	U251	PC-3	K-562	HCT-15	MFC-7	SKLU-1	MDA-MB-231	HGF
**1**	100	100	100	80.93	94.22	70.28	100	13.68
**2**	89.16	100	85.43	73.26	100	82.06	100	2.65
**3**	100	100	100	75.20	100	61.04	100	5.90
**4**	81.78	92.56	100	79.16	82	71	96.64	2.54
Ibuprofen	3.61	7.5	6.3	6.0	19.63	5.18	18.67	9.52
Cisplatin	87.49	42.65	79.15	32.42	52.58	81.35	43.29	ND

ND—None Detected.

**Table 2:** In vitro results of conjugates of ibuprofen against different tumor cell lines and normal HGF fibroblasts expressed as IC_50_ in μM.

Cell Line/(μM ± SD)								
Sample	U251	PC-3	K-562	HCT-15	MFC-7	SKLU-1	MDA	HGF
**1**	3.1 ± 0.1	19.9 ± 1.5	7.3 ± 0.6	11.0 ± 0.7	4.2 ± 0.3	3.6 ± 0.4	6.8 ± 0.9	32.3 ± 4.3
**2**	6.7 ± 0.6	15.9 ± 1.4	3.4 ± 0.8	11.3 ± 0.7	7.9 ± 0.7	12.9 ± 1.1	3.6 ± 1.5	22.3 ± 4.3
**3**	3.3 ± 0.2	4.4 ± 0.3	5.2 ± 0.5	5.9 ± 0.2	5.5 ± 0.4	5.9 ± 0.1	4.3 ± 0.4	20.3 ± 4.3
**4**	3.5 ± 0.5	11.4 ± 0.2	3.0 ± 0.5	6.3 ± 0.4	3.3 ± 0.3	4.2 ± 0.7	3.1 ± 0.5	Not active
Cisplatin	10.8 ± 0.7	17.5 ± 1.4	1.2 ± 0.08	12.3 ± 0.8	17.4 ± 0.8	12.2 ± 0.9	11.2 ± 0.9	12.2 ± 0.9

IC_50_ values are presented as the mean ± SD (standard deviation) from five separate experiments.

## Supplementary Materials

The following are available online at http://www.mdpi.com/2079-4991/7/7/163/s1, Figures S1–S12: full characterization of all the conjugates (^1^H, ^13^C and mass spectrometry); Table S1: elemental analysis of all the conjugates.
