# Supplementary material for: Synthesis, Characterization, and Nanomedical Applications of Conjugates between Resorcinarene-Dendrimers and Ibuprofen
Source: Nanomaterials (Basel). 2017 Jun 30;7(7):163. doi: 10.3390/nano7070163 (PMC5535229; doi:10.3390/nano7070163)

Suplemmentary material

In vitro Cytotoxicity Activity of Resorcinarene-Dendrimer Conjugates of Ibuprofen

Luis D. Pedro-Hernández[1], Elena Martínez-Klimova[2], Sandra Cortez-Maya[1], Sonia Mendoza-Cardozo[1], Teresa Ramírez-Ápan[1], Marcos Martínez-García[1*].

Content

Figure 1. ^{1}H NMR spectrum of the compound **1**

Figure 2. ^{13}C NMR spectrum of the compound **1**.

Figure 3. Mass spectrum of the compound **1.**

Figure 4. ^{1}H NMR spectrum of the compound **2**.

Figure 5. ^{13}C NMR spectrum of the compound **2**.

Figure 6. Mass spectrum of the compound **2.**

Figure 7. ^{1}H NMR spectrum of the compound **3**.

Figure 8. ^{13}C NMR spectrum of the compound **3**.

Figure 9. Mass spectrum of the compound **3.**

Figure 10. ^{1}H NMR spectrum of the compound **4**.

Figure 11. ^{13}C NMR spectrum of the compound **4**.

Figure 12. Mass spectrum of the compound **4.**

Table 1. Elemental analysis of all the conjugates **1-4**.

General procedures for synthesis of resorcinarene-PAMAM-dendrimer-conjugates of ibuprofen

Compounds **1-4** (0.0525 mmol) was dissolved in methanol (40 mL) and heated at 80°C. After 20 min, ibuprofen (0.63 mmol) was added in methanol. The mixture was stirred and heated at 120 °C for 24 h. The solvent was evaporated and the resulting solid was dissolved in metanol and precipitated by EtOAc.

Compound 1. Brown solid (0.170 g, 95%). mp. > 300 °C. UV-vis MeOH (λnm): 283, 205. IR (KBr, cm^{-1}). 3390, 3059, 2921, 2851, 1665, 1611, 1581, 1535, 1499, 1439, 1404, 1360, 1283, 1187, 1123, 1101, 1055, 910, 820, 719, 674, 571. ^{1}H NMR (300 MHz, $CDCl_3$) $\delta_{(ppm)}$: 7.23 (d, 16H, *J*= 7.8 Hz, Ar), 7.06 (d, 16H, *J*= 7.5 Hz, Ar), 6.42 (br, 4H, Ar), 5.94 (br, 16H, NH), 4.70 (br, 4H, CH), 3.59 (br, 8H, CH), 2.97 (s, 32H, CH_2-NH), 2.42 (d, 16H, *J*= 7.2 Hz, CH_2), 1.85 (m, 8H, CH), 1.44 (d, 24H, *J*= 7.4 Hz, CH_3), 1.24 (s, 80H, CH_2), 0.88 (br, 60H, CH_3). ^{13}C NMR (75 MHz, $CDCl_3$) $\delta_{(ppm)}$: 180.2 (C=O, 1), 169.8 (C=O, 2), 153.2 (Ar-O), 139.3 (Ar_{ipso}), 139.2 (Ar_{ipso}), 128.4 (Ar), 126.5 (Ar), 67.7 (CH_2-O), 46.6 (CH), 44.4 (CH_2), 38.3 (CH_2), 36.5 (CH), 31.3 (CH_2), 29.6 (CH), 29.2 (CH_2), 29.1 (CH_2), 28.7 (CH_2), 27.9 (CH_2), 22.0 (CH_2), 21.5 (CH_3), 18.2 (CH_3), 13.3 (CH_3). MS MALDI-TOF (*m/z*): 3410.3. Anal. Calcd. for $C_{208}H_{304}N_{16}O_{24}$: C. 73.20, H. 8.98, N. 6.57 %. Found: C. 73.21, H. 8.98, N. 6.58 %.

Compound 1

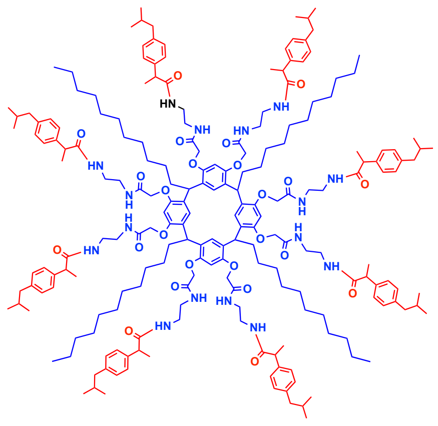

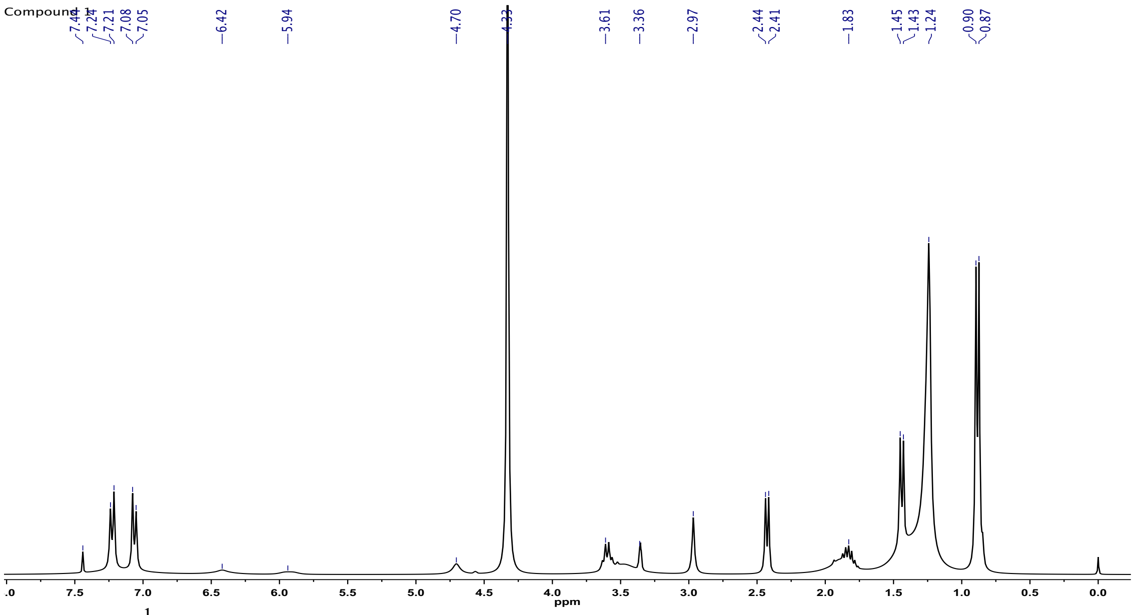

Figure 1. ^{1}H NMR spectrum of the compound **1**.

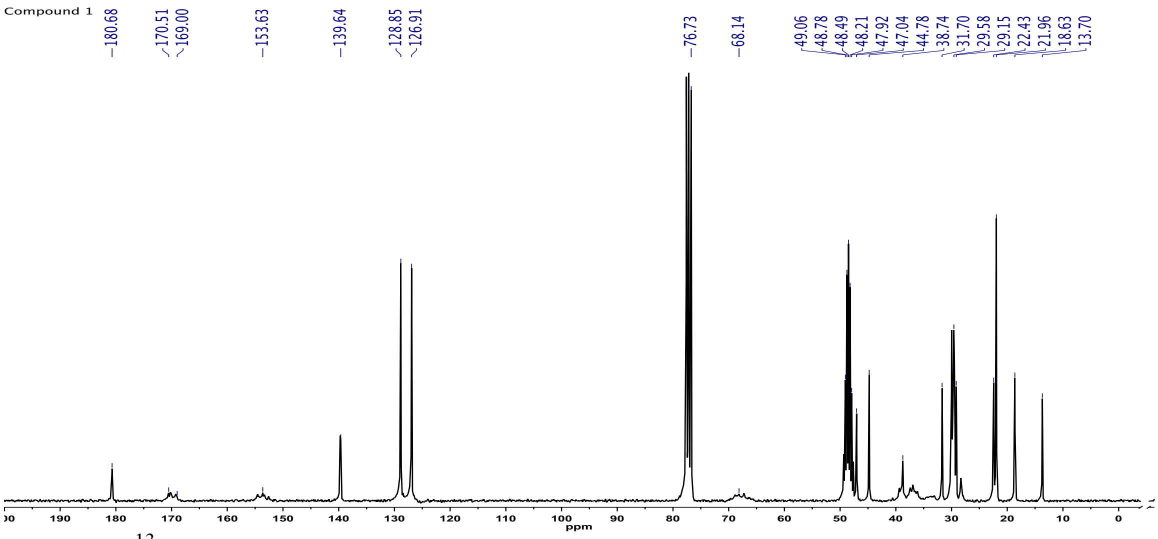

Figure 2. ^{13}C NMR spectrum of the compound **1**.

Registro Dibu G1.0 160912-mgm-03
Matriz Acido 2,5-Dihidroxibenzoico 1/5

Laboratorios de Servicios Analiticos
Instituto de Quimica UNAM

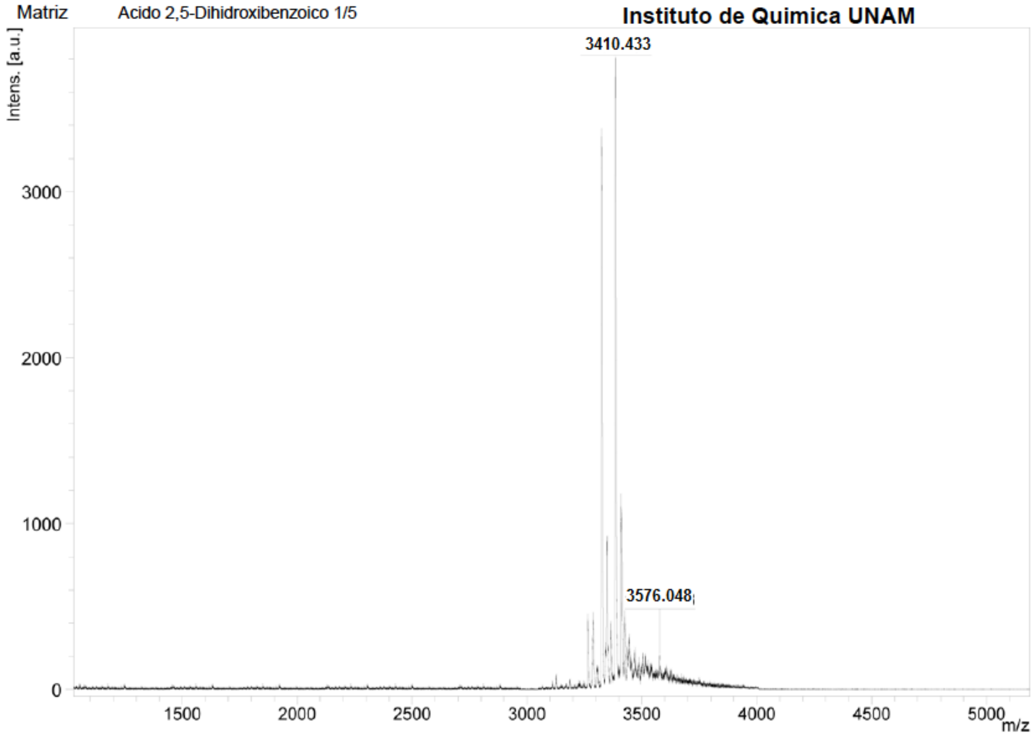

Acquisition Parameter

Date of acquisition	2016-09-14T16:11:45.937-05:00
Acquisition method name	D:\Methods\flexControlMethods\LNM_UNAM\LP_PepMix.par
Aquisition operation mode	Linear
Voltage polarity	POS
Number of shots	200
Name of spectrum used for calibration	
Calibration reference list used	

Instrument Info

User	UNAM
Instrument	FLEX-PC
Instrument type	microflex

D:\data\LSA\Dibu G1.0\0_B8\1

Bruker Daltonics flexAnalysis printed: 9/21/2016 5:00:38 PM

Figure 3. Mass spectrum of the compound **1**.

Compound 2. Brown solid (0.169 g, 90%). mp. > 300 °C. UV-vis CH_2Cl_2 (nm): 284, 235. IR (KBr, cm^{-1}): 3392, 3057,3024, 2952, 2921, 2857, 1669, 1610, 1535, 1500, 1451, 1385, 1363, 1264, 1182, 1112, 1057, 909, 884, 851, 785, 732, 698, 630, 563, 512. ^1H NMR (300 MHz, $CDCl_3$) $\delta_{(ppm)}$: 7.05-7.22 (m, 52H, Ar), 6.61 (br, 4H, Ar), 4.50 (br, 4H, CH), 4.20 (br, 16H, CH_2), 3.59 (m, 8H, CH), 2.98 (br, 32H, CH_2-NH), 2.79 (br, 16H, CH_2), 2.39 (d, 16H, *J*= 6.9 Hz, CH_2), 1.80 (m, 8H, CH), 1.43 (d, 24H, *J*= 6.6 Hz, CH_3), 0.86 (d, 48H, *J*= 6.6 Hz, CH_3). ^{13}C NMR (75 MHz, $CDCl_3$) $\delta_{(ppm)}$: 178.3 (C=O), 168.8 (C=O), 152.6 (Ar-O), 141.9 (Ar_{ipso}), 139.8 (Ar_{ipso}), 139.1 (Ar_{ipso}), 128.9 (Ar), 128.0 (Ar), 127.1 (Ar), 126.1 (Ar), 125.4 (Ar), 68.9 (CH_2-O), 45.9 (CH), 44.8 (CH_2), 37.4 (CH_2), 36.1 (CH), 34.1 (CH_2), 29.9 (CH), 22.2 (CH_3), 18.8 (CH_3). MS MALDI-TOF (*m/z*): 3209.74. Anal. Calcd. for $C_{196}H_{248}N_{16}O_{24}$: C. 73.29, H. 7.78, N. 6.98 %. Found: C. 73.29, H. 7.79, N. 6.98 %.

Compound 2

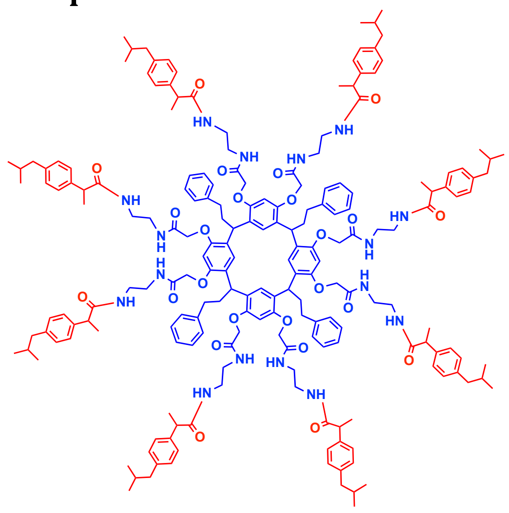

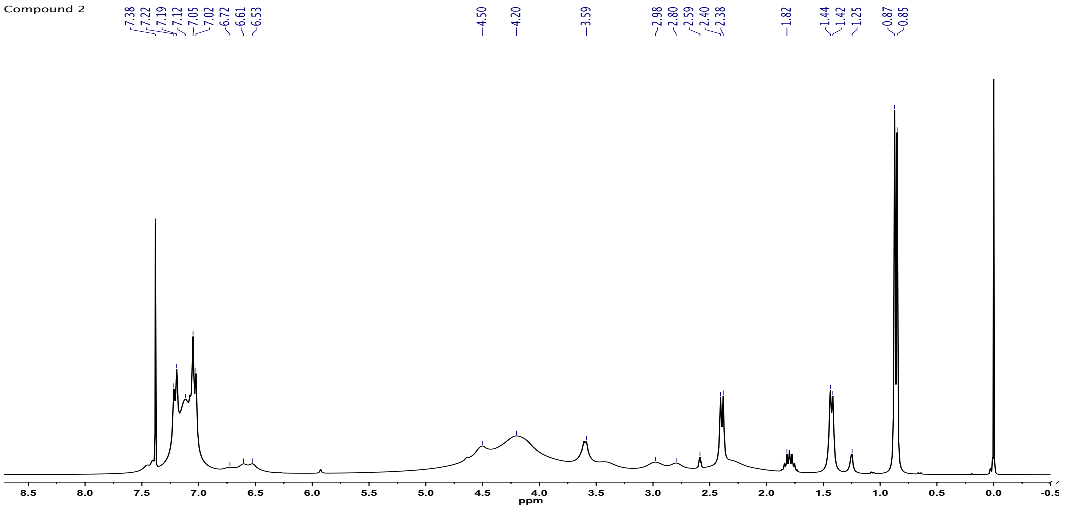

Figure 4. ^{1}H NMR spectrum of the compound **2**.

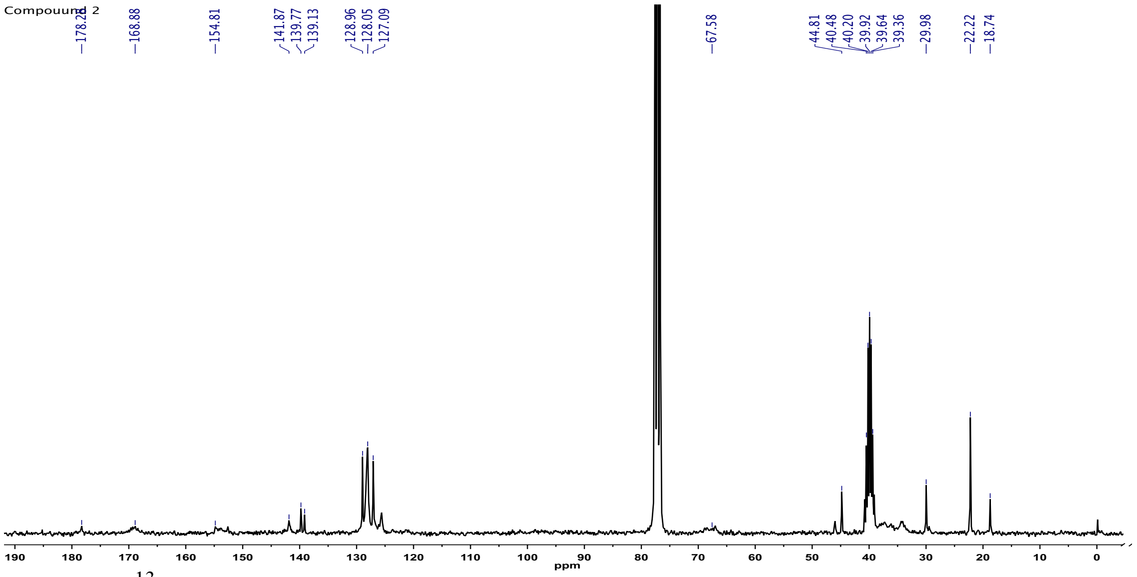

Figure 5. ^{13}C NMR spectrum of the compound **2**.

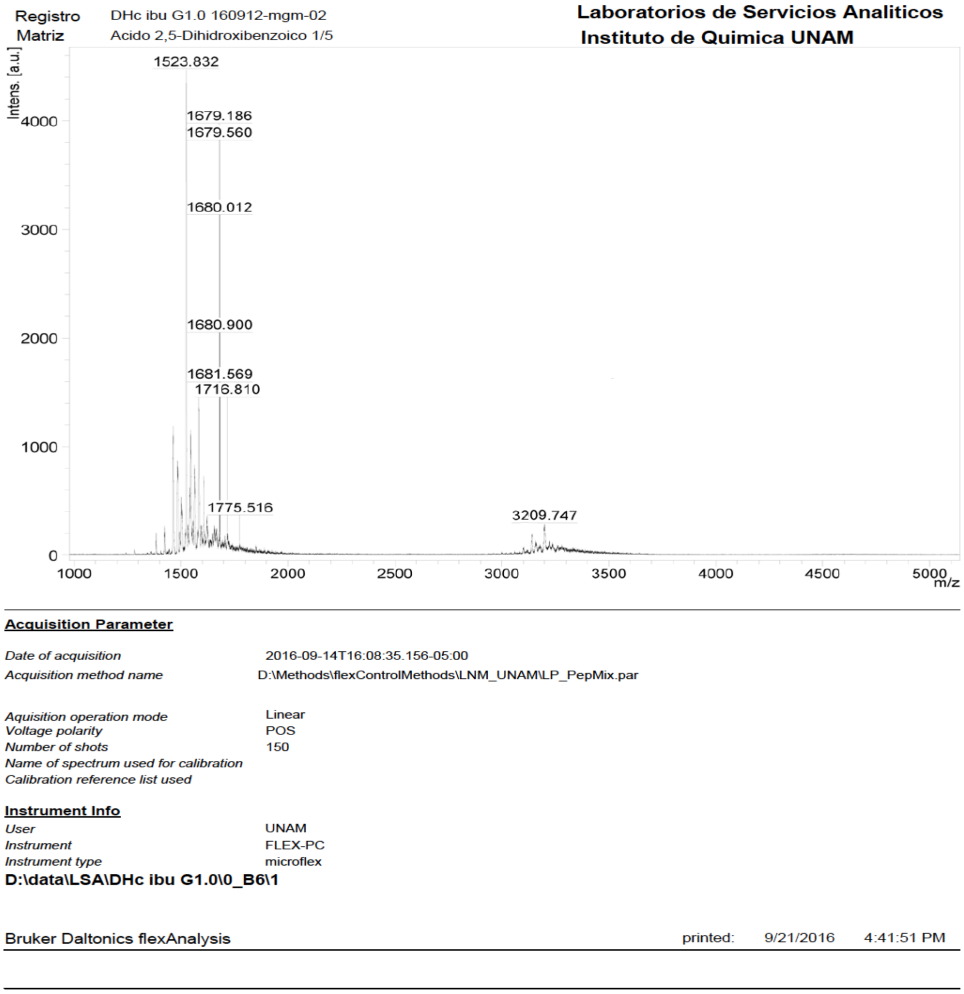

Figure 7. Mass spectrum of the compound **2.**

Compound 3. Brown solid (0.127 g, 70%). mp. > 300 °C. UV-vis CH_2Cl_2 (nm): 232. IR (KBr, cm^{-1}): 3394, 3273, 2852, 2922, 2852, 1654, 1561, 1511, 1457, 1406, 1363, 1280, 1207, 1143, 1118, 1064, 893, 848, 788, 731, 700, 597, 546, 432. ^{1}H NMR (300 MHz, $CDCl_3$) $\delta_{(ppm)}$: 7.23 (s, 32H, Ar), 7.07 (s, 32H, Ar), 6.53 (br, 4H, Ar), 5.91 (br, 40H, NH), 4.71 (br, 4H, CH), 4.57 (br, 16H, CH_2-O), 3.59 (br, 16H, CH), 2.99 (br, 80H, CH_2-NH), 2.72 (br, 48H, CH_2-N), 2.41 (d, 16H, J = 6.0 Hz, CH_2), 1.82 (m, 8H, CH), 1.43 (br, 48H, CH_3), 1.23 (s, 80H, CH_2), 0.88 (br, 108H, CH_3). ^{13}C NMR (75 MHz, $CDCl_3$) $\delta_{(ppm)}$: 181.4 (C=O), 173.8 (C=O), 169.6 (C=O), 154.3 (Ar-O), 140.9 (Ar_{ipso}), 139.8 (Ar_{ipso}), 129.2 (Ar), 127.5 (Ar), 67.7 (CH_2-O), 48.2 (CH), 45.2 (CH_2), 38.6 (CH_2), 37.5 (CH), 32.1 (CH_2), 29.9 (CH_2), 29.5 (CH), 22.8 (CH_2), 22.5 (CH_3), 19.6 (CH_3), 13.3 (CH_3). MS MALDI-TOF (*m/z*): 6740.5. Anal. Calcd. for $C_{392}H_{592}N_{48}O_{48}$: C. 69.81, H. 8.88, N. 9.94 %. Found: C. 68.91, H. 8.88, N. 9.92 %.

Compound 3

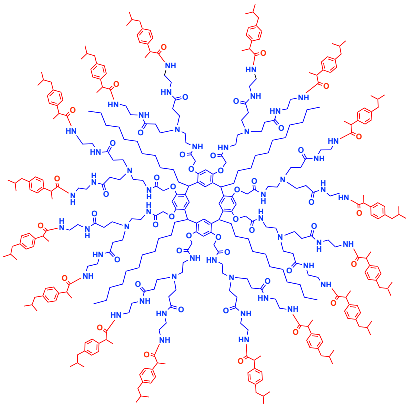

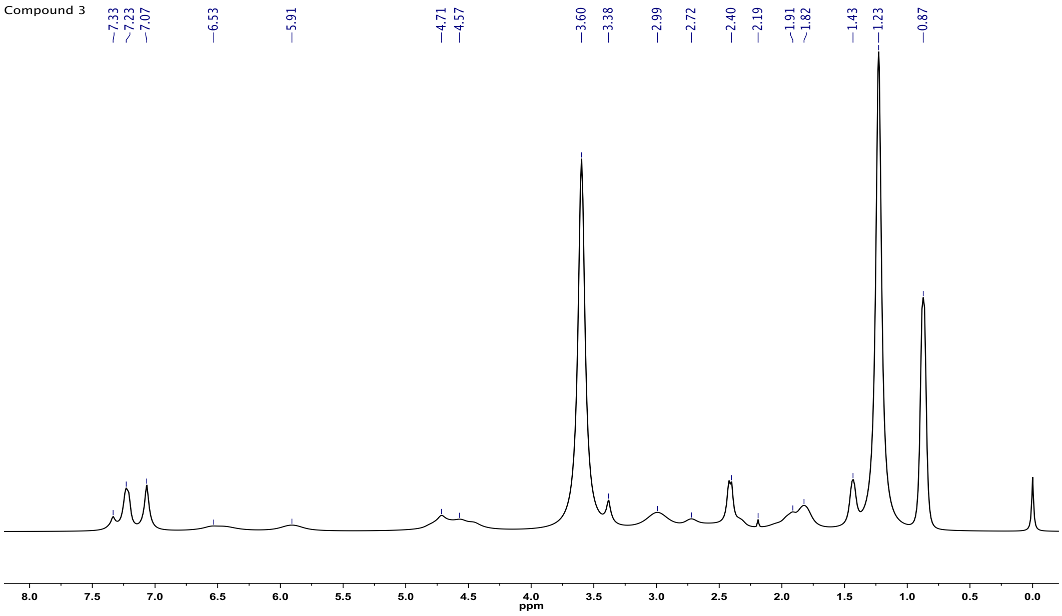

Figure 8. ^{1}H NMR spectrum of the compound **3**.

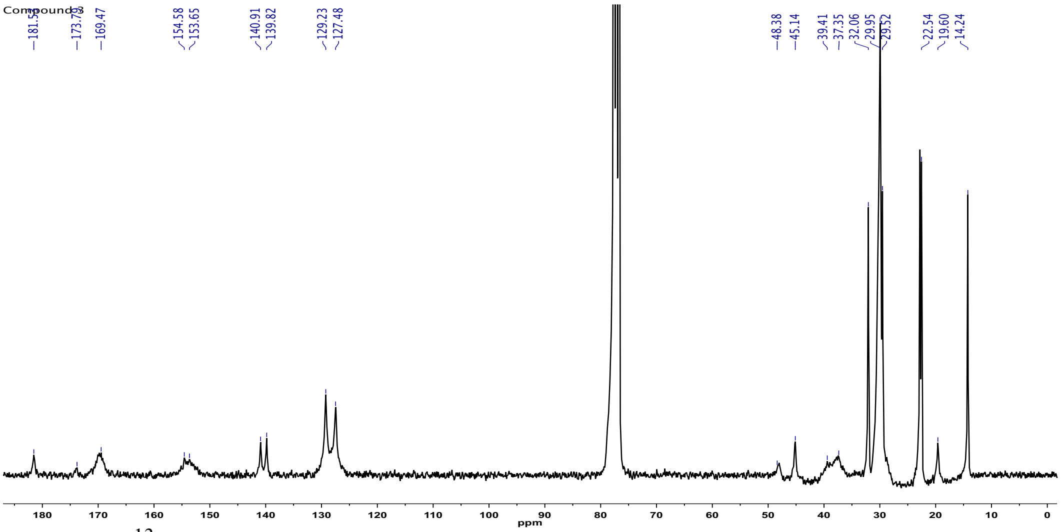

Figure 9. ^{13}C NMR spectrum of the compound **3**.

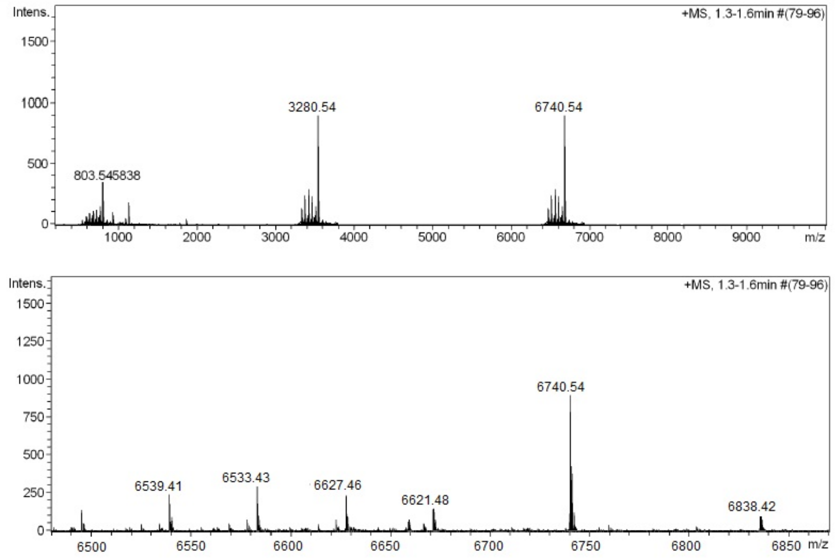

Mass Spectrum SmartFormula Report

Analysis Info

Analysis Name	D:\Data\Dr. Marcos Martínez\Núm. de Reg. 1938.d	Acquisition Date	10/16/2015 10:44:43 AM
Method	tune_high.m	Operator	BDAL@DE
Sample Name	LPRD	Instrument / Ser#	micrOTOF 10255
Comment	Dr. Marcos Martínez García<br>Disolvente: Acetato de Etilo<br>micrOTOF II ESI<br>No. Reg. 1938<br>Realizó: M. en C. Lizbeth Triana Cruz<br>16-10-2015		

Acquisition Parameter

Source Type	ESI	Ion Polarity	Positive	Set Nebulizer	0.3 Bar
Focus	Not active	Set Capillary	4200 V	Set Dry Heater	180 °C
Scan Begin	50 m/z	Set End Plate Offset	-500 V	Set Dry Gas	4.0 l/min
Scan End	10000 m/z	n/a	n/a	Set Divert Valve	Waste

Bruker Compass DataAnalysis 4.0 printed: 10/16/2015 2:35:24 PM Page 1 of 2

Figure 10. Mass spectrum of the compound **3.**

Compound 4. Brown solid (0.130 g, 70%). mp. > 300 °C. UV-vis CH_2Cl_2 (nm): 419, 236. IR (KBr, cm^{-1}): 3274, 3083, 3059, 2953, 2853, 1663, 1559, 1457, 1402, 1384, 1363, 1283, 1249, 1185, 1115, 1063, 848, 785, 753, 732, 699, 669, 585, 548. ^{1}H NMR (300 MHz, $CDCl_3$) $\delta_{(ppm)}$: 7.04-7.23 (m, 84H, Ar), 6.49 (br, 4H, Ar), 4.60 (br, 4H, CH), 3.41 (br, 16H, CH), 2.99 (br, 80H, CH_2-NH), 2.75 (br, 48H, CH_2-N), 2.59 (br, 32H, CH_2-N), 2.41 (d, 32H, J = 6.0 Hz, CH_2), 1.80 (br, 16H, CH), 1.44 (br, 48H, CH_3), 0.87 (d, 96H, J = 6.3 Hz, CH_3). ^{13}C NMR (75 MHz, $CDCl_3$) $\delta_{(ppm)}$: 180.3 (C=O, 1), 173.6 (C=O, 2), 168.8 (C=O, 3), 153.5 (Ar-O), 141.2 (Ar_{ipso}), 139.4 (Ar_{ipso}), 139.1 (Ar_{ipso}), 128.3 (Ar), 127.6 (Ar), 127.4 (Ar), 126.4 (Ar), 125.1 (Ar), 67.8 (CH_2-O), 53.0 (CH_2-N), 50.7 (N-CH_2), 44.2 (CH_2), 38.8 (CH_2), 36.8 (CH_2), 35.6 (CH), 33.7 (CH_2), 32.9 (CH_2), 29.5 (CH), 21.4 (CH_3), 18.2 (CH_3). MS MALDI-TOF (m/z): 6540.1. Anal. Calcd. for $C_{380}H_{536}N_{48}O_{48}$: C. 69.74, H. 8.25, N. 10.27 %. Found: C. 69.72, H. 8.25, N. 10. 29 %.

Compound 4

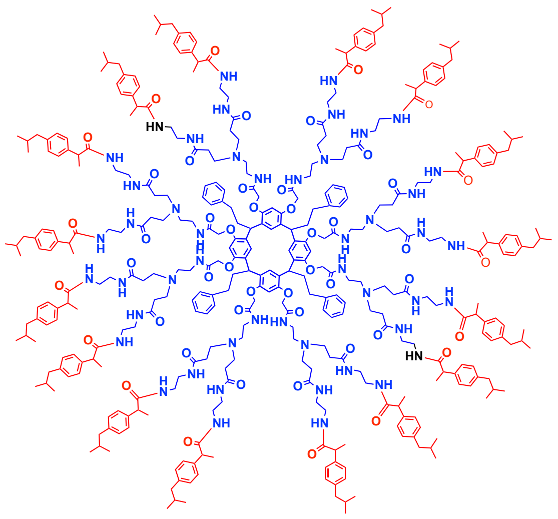

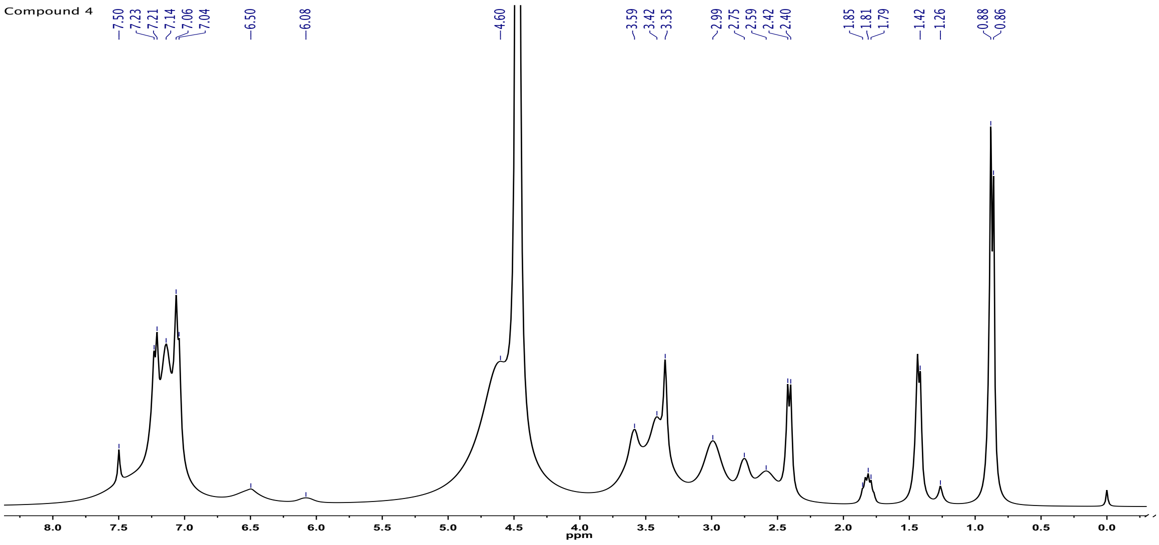

Figure 11. ^{1}H NMR spectrum of the compound **4**.

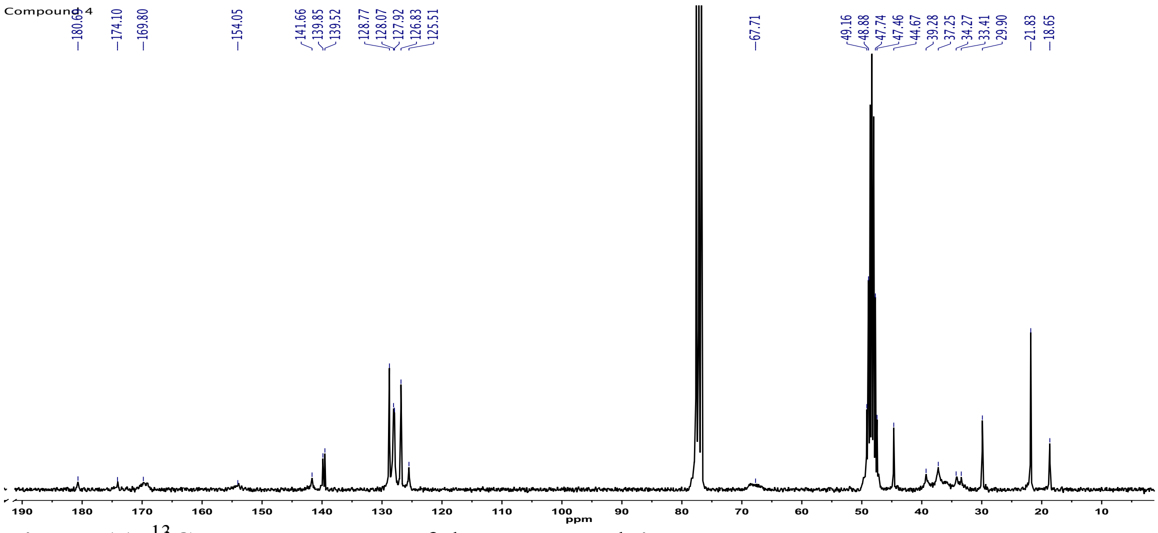

Figure 11. ^{13}C NMR spectrum of the compound **4**.

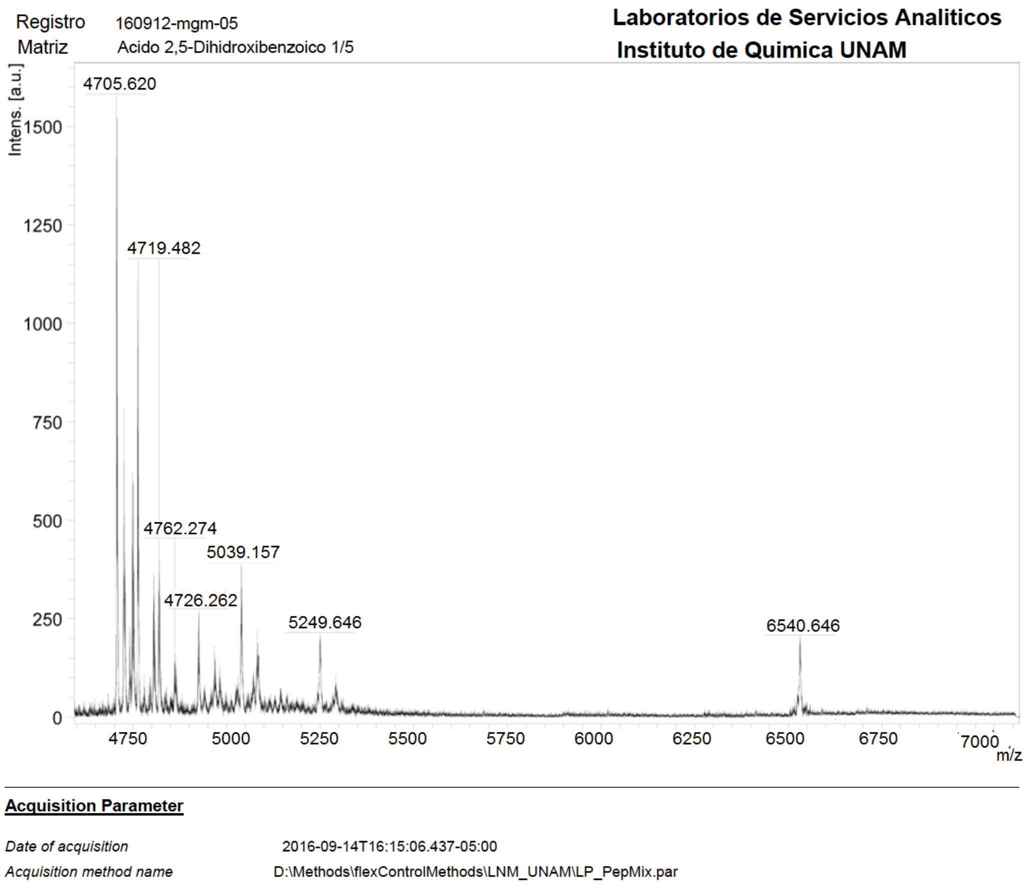

Figure 12. Mass spectrum of the compound **4.**

Elemental analysis of the compounds

Instituto de Química, UNAM
Laboratorios de Servicios Analíticos

Laboratorio de A1.402sis Elemental por Combustión

INFORME DE ANÁLISIS

Investigador solicitante: Dr. Marcos Martínez García

No. de registro	Clave de la muestra	Peso [mg]	N [%]	C [%]	H [%]	S [%]	Fecha de análisis
272	1	1.860	6.58	73.21	8.98	---	16-09-2016
273	2	1.373	6.98	73.29	7.79	---	16-09-2016
274	3	1.855	9.92	68.91	8.88	---	16-09-2016
275	4	1.402	10.29	69.72	8.25	---	16-09-2016

Equipos:

Analizador elemental, marca Thermo Scientific, modelo Flash 2000.
Temperatura del horno: 950 ºC.

Microbalanza, marca Mettler Toledo, modelo XP6.

Control de calidad:

Se utilizó el material de referencia metionina como estándar de verificación, marca Thermo Scientific con número de certificado 232092. El promedio de los valores obtenidos es: N = 9.46 %, C = 40.41 %, H = 7.51 % y S = 21.63 %. Los valores certificados son: N = 9.35 %, C = 40.35 %, H = 7.48 % y S = 21.49 %.

Responsable del análisis: *Q. María de la Paz Orta Pérez*

Fecha de informe: 17 de septiembre de 2016.

Microscopy image of PC-3 cells

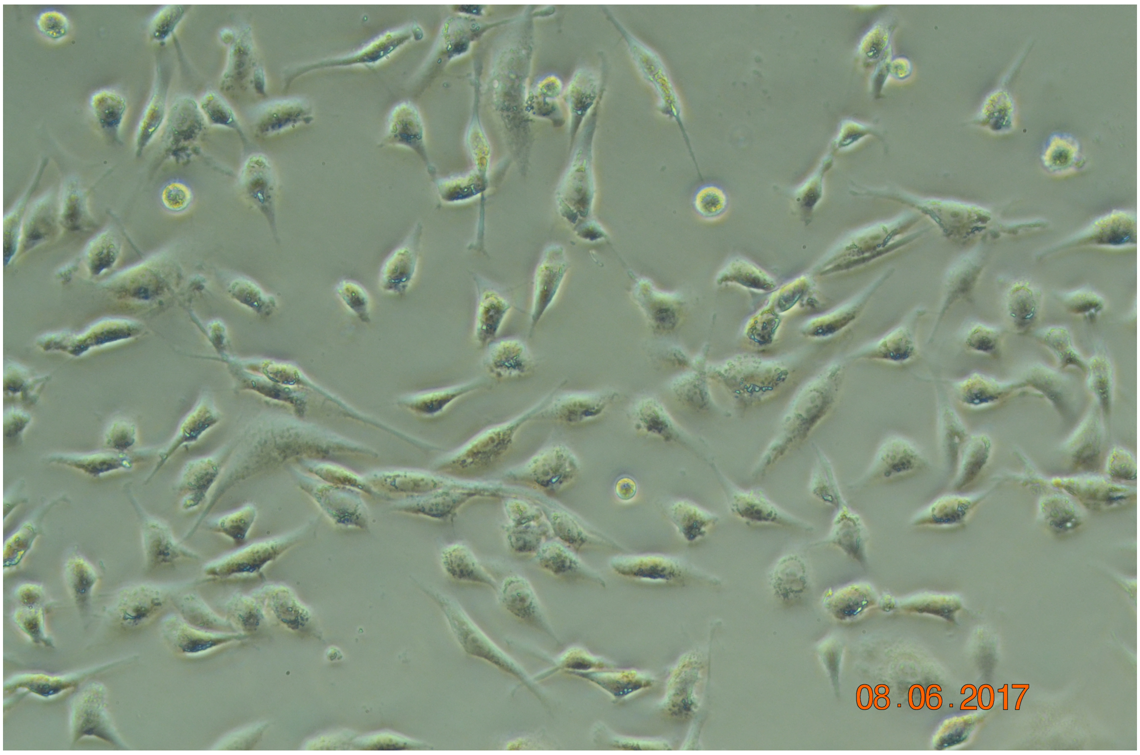

Microscopy image of PC-3 cells with conjugate **4**

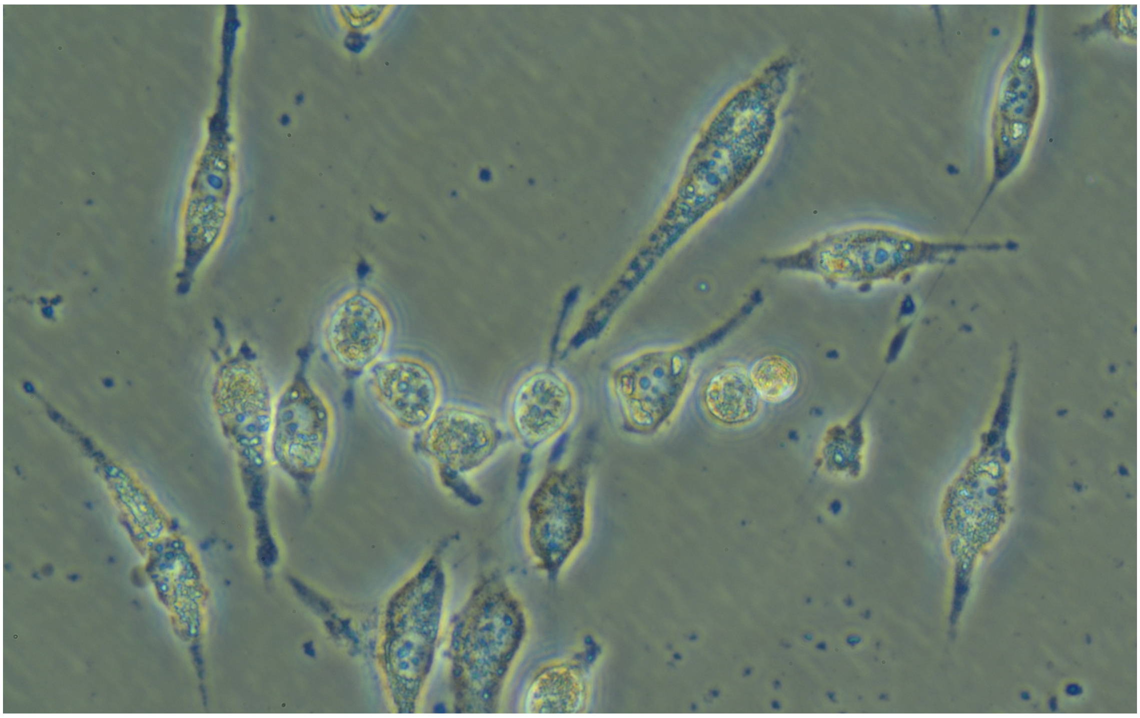

Microscopy image fluorescence of PC-3 cells with conjugate **4**

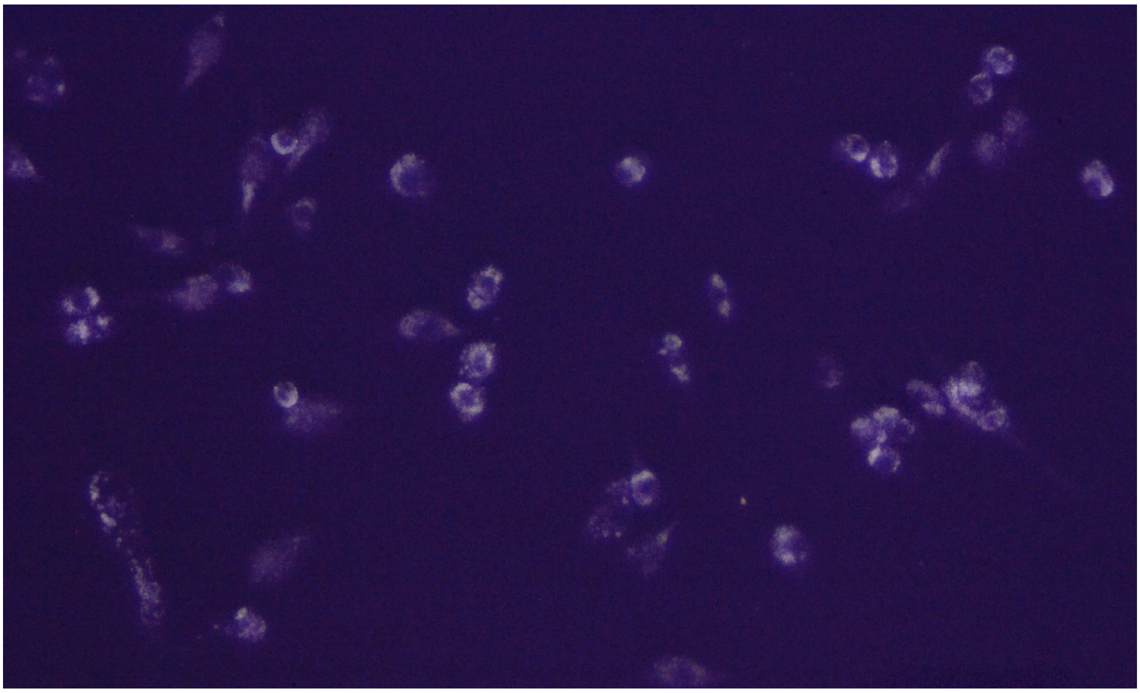

Supplement: Supplementary file 1 [file nanomaterials-07-00163-s001.pdf]
